# Manipulating Engram Histone Acetylation Alters Memory Consolidation

**DOI:** 10.1002/hipo.70041

**Published:** 2025-11-03

**Authors:** Lisa Watt, Liliane Glauser, Davide M. Coda, Johannes Gräff

**Affiliations:** ^1^ Laboratory of Neuroepigenetics, Brain Mind Institute, School of Life Sciences Ecole Polytechnique Fédérale Lausanne (EPFL) Lausanne Switzerland; ^2^ Synapsy Research Center for Neuroscience and Mental Health Research Ecole Polytechnique Fédérale Lausanne (EPFL) Lausanne Switzerland

**Keywords:** dentate gyrus, engram, epigenetic memory, fear learning, HAT/HDAC, histone acetylation

## Abstract

The epigenome provides the brain with a nucleus‐templated form of plasticity that is of fundamental importance for learning and memory. Of the various types of epigenetic modifications, elevations in histone acetylation have been shown to accompany learning and memory, yet the role this epigenetic modification plays within specific cell populations has remained unexplored. Here, we developed a genetic approach to manipulate whole‐genome histone acetylation in memory‐bearing neuronal ensembles. By showing that an increase in histone acetylation promotes fear memory recall, while its downregulation has the opposite effect, we revealed the existence of a functional relationship between histone acetylation and memory expression within memory‐bearing engram cells.

## 1 Introduction

There is a substantial body of evidence that neuronal histone acetylation—an epigenetic modification that generally promotes chromatin accessibility—accompanies neuronal activation and memory formation across multiple brain areas including the hippocampus (Alarcón et al. 2004; Levenson et al. 2004; Guan et al. 2009; Gräff and Tsai 2013; Halder et al. 2016; Fernandez‐Albert et al. 2019). In parallel, several studies reported enhanced cognitive performance upon systemically increasing histone acetylation with pharmacological interventions that stimulate histone acetyltransferases (HATs) or inhibit histone deacetylases (HDACs), the epigenetic machineries responsible for acetyl group deposition and removal, respectively (Benito et al. 2015; Burns et al. 2022; Chatterjee et al. 2018; Fischer et al. 2007; Guan et al. 2009; Gräff et al. 2014; Levenson et al. 2004; McQuown et al. 2011). These advancements notwithstanding, the extent to which histone acetylation causally contributes to the formation, storage, and retrieval of specific memories in defined brain cell populations, as well as the molecular mechanisms underlying it, remains to be deciphered (Coda and Gräff 2024). In our lab, we recently showed that neurons with a high degree of chromatin relaxation prior to encoding are preferentially selected for storing learned information, and thus for becoming part of a bona fide memory‐related cell population (Santoni et al. 2024), the so‐called memory engram (Josselyn et al. 2015; Josselyn and Tonegawa 2020). Here we now asked whether, after encoding, histone acetylation‐driven chromatin plasticity within engram cells plays a role in facilitating the retrieval of newly acquired memories.

## 2 Results

To address this question, we set out to manipulate histone acetylation levels within learning‐induced engram cells by combining the overexpression of HATs or HDACs with a *cFos*‐based labelling system of neuronal activity (Reijmers et al. 2007) in conjunction with a contextual fear conditioning (CFC) memory task. First, to experimentally increase histone acetylation, we generated a lentivirus carrying the HAT CREB‐binding protein (CBP) under the control of a doxycycline (DOX)‐OFF controllable tetracycline response element (TRE), and stereotaxically delivered this construct into the dentate gyrus (DG) of cFos‐tTA mice. In these animals, learning‐induced neuronal activity—via the *cFos* promoter—drives the expression of the tetracycline‐controlled transactivator (tTA), which only binds the TRE element in the absence of DOX.

**FIGURE 1:**
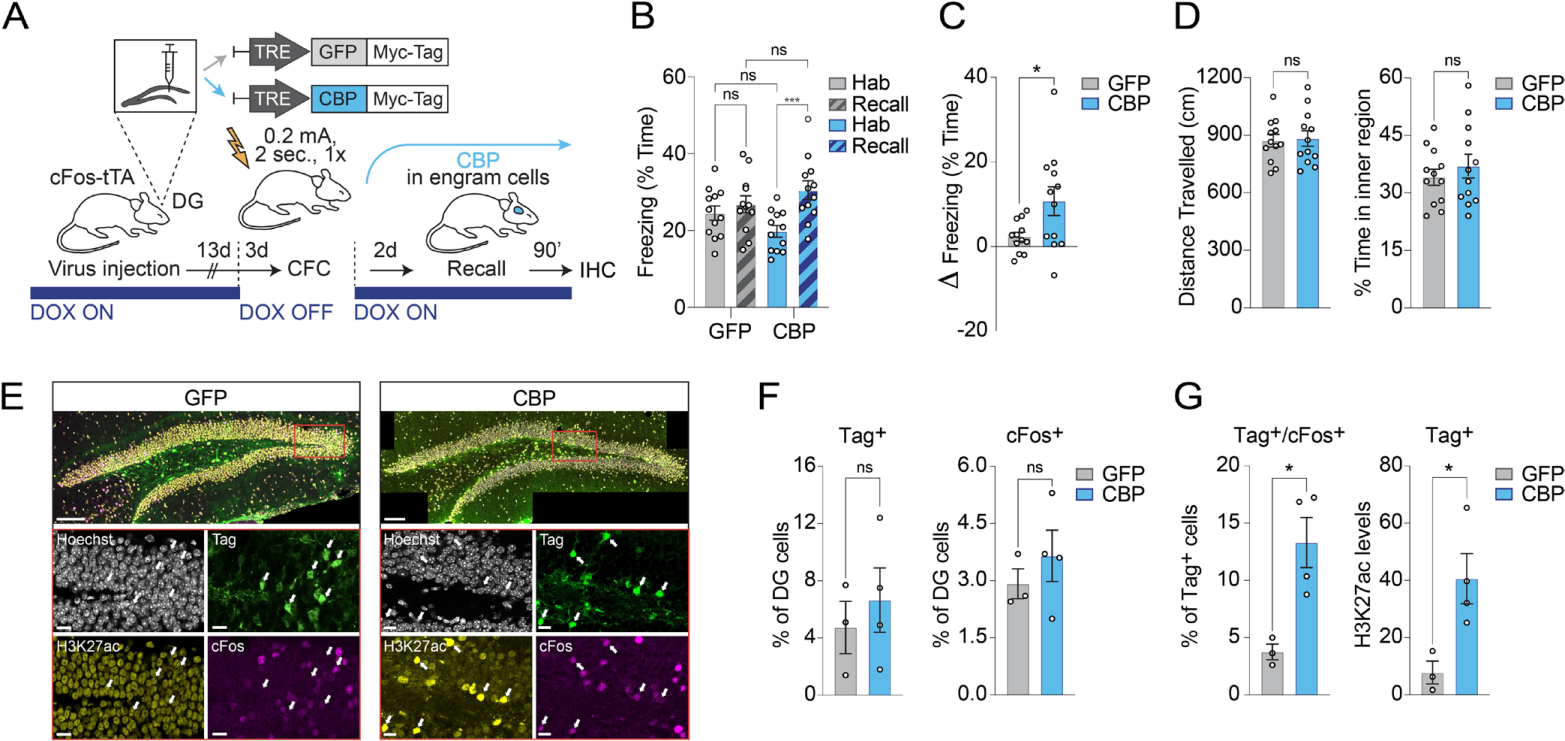
Post‐encoding upregulation of histone acetylation in engram cells enhances fear memory recall. (A) Schematic (top) and experimental design (bottom) for overexpressing the HAT CBP in engram cells of cFos‐tTA mice. For abbreviations, see text. (B, C) Animals injected with CBP (*n* = 12) showed improved memory recall compared to those injected with GFP (*n* = 12) when comparing the pre‐conditioning habituation phase (hab) to the recall phase. Data are means ± SEM compared either by (B) two‐way ANOVA with Sidak's and Fisher's post hoc tests, or by (C) a one‐tailed, unpaired t test. (D) For mice in B and C, displayed are the average distance traveled and the time spent in the inner region of the conditioning chamber during the three‐minute habituation phase of the behavioral conditioning. Data were compared by a two‐tailed, unpaired t test (ns, not significant) and plotted as means ± SEM. (E) Representative confocal images of DG nuclei stained for Hoechst (gray), Myc‐Tag (green), H3K27ac (yellow), and cFos (magenta). Note that the expression of the viral constructs is largely confined to the DG granular cell layer. Arrows indicate neurons positive for Myc‐Tag. Scale bars, 100 μm (top) and 20 μm (bottom). (F, G) Quantification of the number of DG cells positive for (left) Myc‐Tag and (right) cFos in a subset of GFP (*n* = 3) and CBP (*n* = 4) brains. For the Myc‐Tag expressing cells only, plotted are (left) the percentage of cFos positive nuclei and (right) the levels of H3K27ac normalized intensity across the two groups. Data are means ± SEM compared by a two‐tailed, unpaired *t* test. *p < 0.05, **p < 0.01, ***p < 0.001, ****p < 0.0001. Statistical values are reported in Table S2.

To assess if engram histone acetylation improves memory formation, cFos‐tTA mice injected with either CBP or GFP as a control were subjected to a subthreshold CFC task, a paradigm that, alone, only results in modest memory performance (Burns et al. 2022; Denny et al. 2014). Animals were taken off DOX 3 days prior to CFC, permitting the expression of CBP or GFP exclusively in the neurons activated by learning, and put on DOX again immediately after CFC to prevent learning‐unrelated TRE expression. Memory performance was assessed 2 days later by re‐exposing the mice to the conditioned context in the absence of the footshock (Figure 1A).

We found that CBP‐overexpressing mice exhibited a significant increase in freezing rates during the recall phase as compared to those during the pre‐conditioning habituation phase, reflecting the learning of an association between the footshock and context. In contrast, this was not the case for the control group of animals, for which the use of the mild CFC protocol was not sufficient to elicit memory formation (Figure 1B). Importantly, normalizing freezing levels during recall to those during habituation (∆ freezing) confirmed that the CBP‐mediated increase in memory expression was not due to alterations in baseline freezing behavior (Figure 1C). Furthermore, to exclude memory‐unrelated effects of the CBP manipulation, we measured locomotor activity (distance traveled) and anxiety‐related behavior (time spent in the inner region of the behavioral apparatus), both of which did not significantly differ between the two groups (Figure 1D).

We then leveraged the presence of a Myc‐Flag tag fused to the respective inserts in the lentiviral constructs to conduct molecular analyses on CBP and GFP brains collected 90 min after the memory retrieval test. As expected, the percentage of tagged DG cells was comparable between the two groups, reflecting an engram labeling efficiency that was in line with values previously reported in similar studies (Figure 1E,F; Tayler et al. 2013; Ryan et al. 2015). Epigenetically, CBP overexpression led to elevated levels of histone 3 lysine 27 acetylation (H3K27), an epigenetic modification known to favor gene transcription (Creyghton et al. 2010; Kouzarides 2007), within the tagged neuronal population (Figure 1E,G). Furthermore, CBP overexpression resulted in to an increased reactivation of the memory ensemble upon memory recall, as revealed by the higher percentage of labeled engram cells that were positive for cFos in the CBP mice compared to the control condition (Figure 1E,G). Instead, the total number of cFos‐expressing cells in the DG was comparable between the two groups of animals, indicating that the size of the recall ensemble was not affected by the manipulation (Figure 1E,F). Together, these results suggest that promoting chromatin relaxation in memory‐encoding neurons by means of CBP‐dependent histone acetylation favors their reactivation during memory retrieval and leads to enhanced contextual fear memory retention.

**FIGURE 2:**
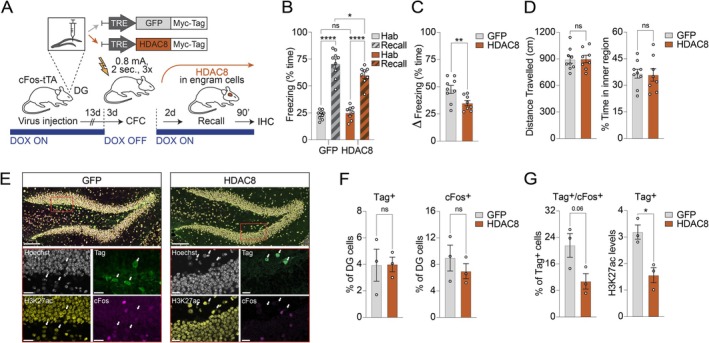
Post‐encoding downregulation of histone acetylation in engram cells reduces fear memory recall. (A) Schematic (top) and experimental design (bottom) for overexpressing the HDAC HDAC8 in engram cells of cFos‐tTA mice. For abbreviations, see text. (B, C) Animals injected with HDAC8 (*n* = 8) showed improved memory recall compared to those injected with GFP (*n* = 9) when comparing the pre‐conditioning habituation phase (hab) to the recall phase. Data are means ± SEM compared either by (B) two‐way ANOVA with Sidak's and Fisher's post hoc tests, or by (C) a one‐tailed, unpaired t test. (D) For mice in B and C, displayed are the average distance traveled and the time spent in the inner region of the conditioning chamber during the three‐minute habituation phase of the behavioral conditioning. Data were compared by a two‐tailed, unpaired t test (ns, not significant) and plotted as means ± SEM. (E) Representative confocal images of DG nuclei stained for Hoechst (gray), Myc‐Tag (green), H3K27ac (yellow), and cFos (magenta). Note that the expression of the viral constructs is largely confined to the DG granular cell layer. Arrows indicate neurons positive for Myc‐Tag. Scale bars, 100 μm (top) and 20 μm (bottom). (F, G) Quantification of the number of DG cells positive for (left) Myc‐Tag and (right) cFos in a subset of GFP (*n* = 3) and HDAC8 (*n* = 3) brains. For the Myc‐Tag expressing cells only, plotted are (left) the percentage of cFos positive nuclei and (right) the levels of H3K27ac normalized intensity across the two groups. Data are means ± SEM compared by a two‐tailed, unpaired *t* test. *p < 0.05, **p < 0.01, ***p < 0.001, ****p < 0.0001. Statistical values are reported in Table S2.

Next, we tested whether decreasing histone acetylation by overexpressing HDAC8, a class I HDAC that catalyzes the deacetylation of histone residues (Castañeda et al. 2017), including H3K27ac (Ha et al. 2014), and leads to reduced transcription (Hu et al. 2000), would trigger the opposite behavioral effect to CBP. To this end, TRE‐HDAC8 or TRE‐GFP constructs were stereotaxically injected into the DG of cFos‐tTA mice (Figure 2A). Since we hypothesized that our manipulation would this time reduce memory expression, we used a stronger CFC protocol to avoid floor effects. With this paradigm, the control group showed high freezing levels during the recall phase, reflecting the formation of a robust fear memory (Figure 2B). On the contrary, we observed that HDAC8‐overexpressing mice froze significantly less than the GFP group, suggesting an HDAC8‐driven reduction in memory expression, which was confirmed when calculating the ∆ freezing (Figure 2B,C). Locomotion and baseline behavior were similar across both groups (Figure 2D). Molecular analyses revealed comparable infection efficiency, but lower levels of H3K27ac in engram cells overexpressing HDAC8 compared to the control GFP‐expressing cells (Figure 2E–G). Finally, the percentage of tagged DG neurons did not significantly differ across conditions, while their re‐activation by recall‐induced cFos was lower in the HDAC8 than in the control group (Figure 2E–G). The latter finding suggests that inducing a closed chromatin conformation by histone hypoacetylation post‐encoding diminishes the probability for a given engram cell to become re‐activated upon re‐exposure to fear‐associated cues.

In summary, these experiments show that altering CBP and HDAC8 levels in neuronal ensembles after learning leads to bidirectional histone acetylation changes and results in modified memory performances. Mechanistically, it is likely that modulating global H3K27ac levels profoundly impacts the engram epigenetic landscape and directly affects their capacity to store and retrieve fear memory information. Neurons activated by learning are known to display increased chromatin accessibility, which has been proposed to functionally bookmark specific genomic sites for rapid transcriptional activation during later memory phases (Marco et al. 2020). In this scenario, we speculate that the CBP‐mediated enhancement of chromatin plasticity upon memory encoding would sharpen such epigenetic priming events, transitioning engram cells into a poised epigenetic state that may favor the recall of the acquired memory. The opposite would occur upon promotion of chromatin compaction in an HDAC8‐dependent manner. To corroborate this hypothesis, it would be important to mechanistically address that the observed effects were specifically driven by acetylation changes and not by the activity of CBP/HDAC8 on non‐histonic substrates.

Our lab has recently shown that prior to learning, CBP‐driven hyperacetylation positively biases a neuron's eligibility to be recruited into the memory trace by epigenetically increasing its excitability and synaptic functions (Santoni et al. 2024). Although in the current study we did not characterize the epi‐transcriptional profile nor the electrophysiological properties of CBP‐overexpressing neurons, it is plausible that an epigenetic mechanism similar to that governing memory allocation may also drive the facilitated reactivation of CBP positive cells we observed upon memory recall. Furthermore, whether learning itself induces histone acetylation changes within engram cells, at which genomic sites and at which histone residues other than K27, remains to be determined. Future work should aim to tease apart the causal effects of H3K27ac alterations and other histone modifications on memory performances, taking advantage of emerging technologies such as locus‐specific CRISPR‐based epigenetic editing (Coda et al. 2025). While these questions await an answer, our results provide for the first time a functional indication that post‐encoding histone acetylation within engram cells is crucial for memory expression.

## 3 Detailed Methods

### 3.1 Molecular Cloning and Lentivirus Production

The CBP and HDAC8 plasmids were obtained in‐house by subcloning into the lentiviral pVLX backbone under control of the TRE promoter the CBP catalytic domain previously characterized in the lab (Santoni et al. 2024), and the HDAC8 portion of the dCas9‐HDAC8 vector (Chen et al. 2019), which was a gift from Dr. Anne West. A full list of the plasmids used in the study is provided in Table S1. Lentiviral vectors were produced as previously described (Sanchez‐Mut et al. 2018).

### 3.2 Animals

All animals and procedures used in this study were approved by the Veterinary Office of the Federal Council of Switzerland under the animal experimentation license VD2808.2. cFos‐tTA male mice were bred in house from the original JAX strain #018306 on a C57Bl/6JR background. Animals were group‐housed in a 12 h light/dark cycle with water and food available ad libitum. DOX (0.2 mg/mL) was given orally through the water supply beginning at least 7 days before the start of the experiment, and only ceased 3 days prior to the session where the tagging window was desired (Khalaf et al. 2018; Lamothe‐Molina et al. 2022; Liu et al. 2012; Redondo et al. 2014; Ryan et al. 2015; Sørensen et al. 2016). DOX was then provided back to the animals as soon as the tagging window was no longer needed. Mice were between 8 and 13 weeks old at the start of the experiments, and all males. All behavioral procedures were performed between 1 and 5 pm local time and animals were randomly assigned to experimental groups.

### 3.3 Virus Injections and Behavioral Procedures

Surgeries were conducted as described previously (Dixsaut and Gräff 2022). For each lentivirus 500 ng, which corresponded to a volume between 200 and 500 nL depending on the individual virus titer, was injected bilaterally. Contextual fear conditioning behavioral experiments were performed using a TSE Multi Conditioning System. CFC encoding consisted of a first 3 min exploration phase, followed by 2 s long foot shocks spaced by 28 s. The number of repetitions of the pause‐shock stage as well as the electrical current varied depending on the experiment (see figures). After the last shock, the animal was left in the chamber for an additional 15 s and brought back to its home cage. The recall phase took place 48 h after encoding and consisted of a 3 min exposure to the same context, without any shock. The movement of the animals was automatically measured using an infrared beam cut detection system (TSE Systems) and freezing was calculated as the absence of movement for more than 500 ms.

### 3.4 Immunohistochemistry, Image Acquisition and Analysis

90 min after the memory recall test, animals were anesthetized with pentobarbital (150 mg/kg) and transcardially perfused with first PBS and then 4% paraformaldehyde (PFA) in PBS. Brains were extracted, post‐fixed overnight in 4% PFA, transferred in a cryoprotectant solution (30% sucrose in PBS) for at least 48 h, and frozen at −80°C. Sections of 20 μm were cut using a cryostat, and processed for immunohistochemistry as previously described (Santoni et al. 2024). Primary and secondary antibodies used are listed in Table S1 alongside their working concentrations.

Images were obtained using an Upright Leica DM6 CS laser scanning confocal microscope at 63× magnification with a resolution of 512 X 512, speed of 400 Hz, airy unit between 0.3 and 0.4 AU and line averaging of three. Channels were acquired from the longest wavelength to the shortest wavelength and acquired one at a time to avoid leakage between the channels with spectrum overlaps assessed with BD spectrum viewer (BD Biosciences). A tilescan of the acquired area was then stitched within the Leica LAS‐X software and analyzed using QuPath (v0.2.3 to v0.4.3) (Bankhead et al. 2017). For each confocal image of a single DG, detected nuclei were first classified as part of the DG granular cell layer or not using a machine‐learning based algorithm developed in house starting from the StarDist library. Next, nuclei were defined as positive for each given channel if their mean signal was higher than a manually pre‐set threshold of detection. To compute H3K27ac levels, for each nucleus H3K27ac signal intensity was normalized by its corresponding Hoechst value and then averaged across the other tagged cells to obtain a single measurement per acquired image. Overall, a total of four images were analyzed for each mouse, and three to four mice per group were compared in each experiment.

### 3.5 Statistics and Reproducibility

Prism 9 (GraphPad) was used to perform statistical analyses. All data are displayed as mean ± standard error mean (SEM), and the statistical test used and the definition of n are described in the figure legends. Statistical analysis details for each figure are reported in Table S2. No statistical methods were used to predetermine sample sizes, but the number of animals used in each experiment is similar to those reported in previously published engram studies. Data distribution was assumed to be normal, even if this was not formally tested. Animals were randomly assigned to experimental groups and age matched.

## Author Contributions

D.M.C. and J.G. designed and conceptualized this study. L.W. carried out the experiments and analyzed the data. L.G. contributed to mouse colony maintenance and lentivirus production. D.M.C. and J.G. wrote the manuscript with comments from all authors.

## Conflicts of Interest

The authors declare no conflicts of interest.

## Supporting information


**Table S1:** List of materials, reagents and software used in this study.

**Table S2:** Statistics summary for all the experiments in this study.

## Data Availability

The data that support the findings of this study are available from the corresponding author upon reasonable request.
